# Nanomaterials on Plant Growth and Stress Adaptation

**DOI:** 10.3390/plants14111651

**Published:** 2025-05-29

**Authors:** Yolanda González-García, Antonio Juárez-Maldonado

**Affiliations:** 1 National Institute of Forestry, Agriculture and Livestock Research, Northwest Regional Research Center, Todos Santos Experimental Field, La Paz 23070, Mexico; 2 Department of Botany, Autonomous Agrarian University Antonio Narro, Saltillo 25315, Mexico; 3 Conahcyt’s National Laboratory of Plant Ecophysiology and Food Security (LANCEVSA), Autonomous Agrarian University Antonio Narro, Saltillo 25315, Mexico

## 1. Introduction

Nanotechnology has been proven to be a useful tool in many fields. Through innovative applications, nanomaterials (NMs) such as nanoparticles and nanocomposites offer unique properties that can positively influence plant growth and adaptation to stress. Nanomaterials can induce responses in plants as soon as they come into contact with cell walls and cell membranes or by internalizing inside the cell. This causes changes at different levels, such as biochemical, genetic, or metabolic levels, which translate into physiological and secondary metabolism modifications that improve the functioning of plants. They can enhance nutrient uptake, improve water retention, and provide protection against environmental stressors such as drought, salinity, and heavy metals, among others [1,2,3]. By harnessing the potential of nanomaterials, we can address global challenges in food security and sustainable agriculture while minimizing the occurrence of adverse environmental effects.

The application of nanomaterials in agriculture is one of the most important tools currently available to address the different stress conditions, both biotic and abiotic, that negatively affect crops. First of all, NMs have a positive effect on several stages of plant growth and development, including germination, but also at biochemical and physiological levels, such as photosynthesis, nutrient absorption, and metabolism in general [1,2,4]. From these positive responses, it is possible to induce tolerance to a variety of stressful conditions to which plants may be exposed, such as drought, salinity, heavy metals, high and low temperatures, pathogens, among others [1]. Some of the specific mechanisms activated by the application of NMs in plants that are fundamental for their participation in tolerance to stress conditions are ROS regulation and antioxidant activity, changes in primary and secondary metabolism, changes in gene expression and signaling pathways, hormonal balance, and osmolyte accumulation, among others [1,2,4].

This Special Issue includes a total of twelve articles, of which seven are original articles and five are reviews, and presents updated information on the application of NMs in different crops, especially with the aim of coping with a variety of stress conditions. These publications demonstrate the importance of nanotechnology applied to agriculture with the goal of inducing tolerance to different stresses. Topics covered in this Special Issue include salt stress, water stress, flooding, and nutrient deficiency. Furthermore, the information presented covers different types of nanomaterials and nanoparticles, both metallic and organic, and is geared toward stimulating plant growth and stress adaptation. All this information definitely contributes to the progress towards a more sustainable agriculture and one that, above all, has a greater capacity to adapt to stress.

## 2. Results

It is clear that the induction of tolerance to different stress conditions via the application of NMs is due to changes at different levels in plants, including genetic, physiological, biochemical, and morphological changes [1,2]. This capacity of NMs is due to their unique properties resulting from their size (<100 nm), which allows them to be absorbed and translocated through plants in different ways than traditional nutrients. As they pass through the different plant structures, they interact with different tissues, cells, and cell organelles, in turn triggering a cascade of responses that can be positive; in excessive amounts, however, NMs can become toxic [2]. In any case, it is the positive responses that are of interest for agriculture.

Therefore, NMs can be used directly as a source of nutrients, since their properties can provide advantages over conventional fertilizers, such as higher efficiency and absorption and translocation capacity, but also stimulate plant growth and development [3]. Morfín-Gutiérrez et al. [5] demonstrated that the application of NMs based on Fe and silicon (Fe_3_O_4_@MCM-48) was efficient to replace the application of conventional Fe fertilizers, with the additional advantage of improving some biochemical parameters such as the content of antioxidant pigments and carotenoids in tomato plants.

For other types of stresses, such as flooding, salinity, and drought, positive effects have been reported for the application of various NMs [6,7,8,9,10]. Indeed, one of the major challenges of agriculture is the problem of soil salinity. Haghmadad Milani et al. [8] showed that application of cerium oxide nanoparticles (CeO_2_ NPs, 25 mg L^−1^) improved the salinity tolerance of spearmint (*Mentha spicata* L.) plants by increasing chlorophyll and carotenoid content, enhancing antioxidant enzyme activities, and reducing MDA and H_2_O_2_ levels. However, the authors observed that high concentrations of these nanoparticles induced negative effects on the plants. Jin et al. [7] applied silica nanoparticles (SiO_2_ NPs) to rice plants (*Oryza sativa* L., Y liangyou 957 [YLY957] and Jingliangyou 534 [JLY534]) subjected to salt stress (0.6% saline solution). Their results showed that the application of SiO_2_ NPs improved the quality of rice yield under high salt stress due to changes at biochemical (chlorophyll content, antioxidant enzyme activities) and morphological (grains per panicle and grain filling rate, leaf area index, dry matter accumulation, and stimulation of root system growth and development) levels. Hafez et al. [9] evaluated the synergistic effect of applying sugarcane bagasse (SCB) and zinc oxide nanoparticles (ZnO NPs) to mitigate the adverse effects of cadmium and salinity on wheat plants. Their results showed that their combined application significantly mitigated the effects of Cd and salinity on soil and wheat plants. They were able to reduce Cd accumulation in plants, and antioxidant enzyme activity was enhanced, resulting in higher grain yield.

Kathirvelan et al. [6] applied zinc oxide nanoparticles (ZnO NPs) and manganese oxide nanoparticles (MnO NPs) via foliar application to drought-stressed maize (*Zea mays* L.) plants. The results obtained suggest that foliar application of these nanoparticles to drought-stressed maize plants can reduce negative effects and significantly improve the grain yield of maize. By contrast, Novikova et al. [10] evaluated the application of boron and cobalt nanoparticles and their putative protective effects on two wheat cultivars under flood induction in a closed system. Their results showed positive effects of the application of these nanoparticles—cobalt and boron nanoparticles enhanced adaptation to stress and improved photosynthetic parameters.

Regarding the induction of tolerance to biotic stress, Alfosea-Simón et al. [11] present a complete review on the use of silver nanoparticles (Ag NPs) to address this problem. The authors describe how Ag NPs have been effective in controlling pathogens both in vitro and ex vitro but also suggest that attention should be paid to their management, since under certain conditions they are also capable of inducing negative responses in plants.

Interestingly, work is also being carried out with organic nanoparticles, which by their nature may have advantages over metal nanoparticles. Tolisano et al. [12] studied the application of lignin nanoparticles (LNs) from pomace as a nanobiostimulant in tomato plants. They found that the application of LNs increased the efficiency of light capture and utilization, pigment content, nitrogen content (NBI), and flavonoids. These results demonstrate that nanotechnology based on organic materials can be a viable option for agriculture that can support the pursuit of sustainability and food security.

## 3. Conclusions

Global agricultural production continuously faces stressors that compromise the food supply. This Special Issue presents various approaches demonstrating how nanomaterials enhance plant species’ responses to abiotic stress factors, including flooding, water shortages, soil and water salinity, heavy metals, and extreme temperatures, as well as biotic stress factors, such as bacterial, fungal, and viral attacks. Thus, this Special Issue provides a thorough overview of the mechanisms by which nanomaterials promote plant growth and adaptation to stress, offering valuable insights and promoting the development of new agricultural products.
